# The Beat

**Published:** 2011-01

**Authors:** Erin E. Dooley

## Report Finds Estimates of Gulf Coast Exposure to Carcinogens Off

The Natural Resources Defense Council reports the FDA underestimated seafood consumption by Gulf Coast residents in developing their June 2010 protocol for determining safe seafood levels of toxic PAHs following the BP *Deepwater Horizon* oil spill.^1^ The FDA used national consumption data, rather than region-specific information and also did not take into account the dietary patterns of subpopulations including children and the region’s large Vietnamese-American population. Gulf Coast shrimp consumption rates were found to range from 3.6 to 12.1 times higher than the FDA estimates.

**Figure:**
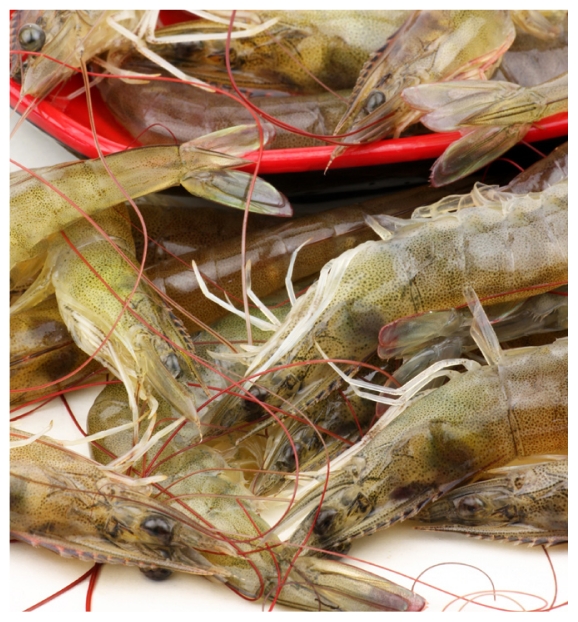
Gulf Coast residents eat an average of two shrimp meals per week, twice the FDA estimate.

## Federal Bedbug Summit in February

On 1–2 February 2011 the Federal Bed Bug Workgroup will sponsor the second national bedbug summit in Washington, DC.^2^ The meeting will be open to the public and accessible via a webinar. The workgroup will review the current bedbug problem and seeks to identify and prioritize actions to manage and control these increasingly prevalent and resistant pests.

**Figure:**
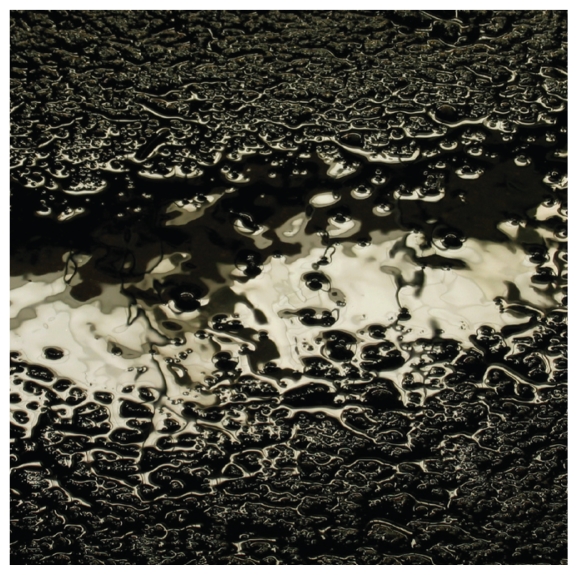
Lakes in cities where coal tar sealant is most commonly used had far higher PAH levels than other lakes.

## Coal Tar Sealant a Significant Lake Pollutant

USGS researchers used a chemical mass-balance model to show that coal tar pavement sealants were the chief source of PAHs flowing into 40 U.S. urban lakes.^3^ Surface water concentrations of PAHs, which are a probable human carcinogen and are toxic to fish and other aquatic life, have been increasing in recent decades. Being able to determine the source of these PAHs will help in the design better ways to manage them. Some U.S. municipalities have already banned coal tar sealants.

## Ford Cottons to Recycling

Ford Motor Company recently announced its 2012 Ford Focus models will use carpet backing and soundproofing materials made from recycled cotton denim.^4^ Cotton production can have a large environmental footprint, and clothing and other textiles represent about 4% of municipal solid waste.^5^ Each car will use an amount of postconsumer cotton equal to the amount in two pair of jeans.^4^

## Greenwashing Update

“Greenwashing” is the term for ads and labels that promise more environmental benefit than they deliver.^6^ The third in a series of reports by TerraChoice Environmental Marketing finds that marketers are getting better at substantiating claims of “greenness” about their products.^7^ The number of self-described green products tallied on shelves increased 73% between 2009 and 2010, with 4.5% of such products making credible claims. In 2007, only 1% of the claims made by surveyed products could be verified. One area where marketing claims have skyrocketed is in products claiming they have no bisphenol A (up 577% over 2009) or no phthalates (up 2,550% over 2009).

**Figure:**
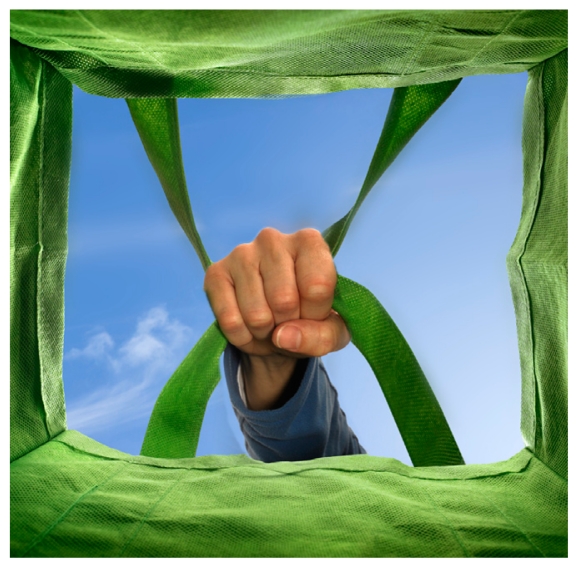
A survey of green product claims found 4.5% to be bona fide, up from 1% in 2007.
